# Identification of cassava quality attributes preferred by Ugandan users along the food chain

**DOI:** 10.1111/ijfs.14878

**Published:** 2020-12-03

**Authors:** Paula Iragaba, Sophia Hamba, Ephraim Nuwamanya, Michael Kanaabi, Ritah Ann Nanyonjo, Doreen Mpamire, Nicholas Muhumuza, Elizabeth Khakasa, Hale Ann Tufan, Robert Sezi Kawuki

**Affiliations:** ^1^ National Crops Resources Research Institute (NaCRRI) P.O. Box 7084 Kampala Uganda; ^2^ National Agricultural Research Laboratories (NARL) P.O. Box 7065 Kampala Uganda; ^3^ International Programs College of Agriculture and Life Sciences B75 Mann Library Ithaca NY 14853 USA

**Keywords:** Adoption, breeding, consumer, end user, *Manihot esculenta*, quality, survey

## Abstract

This study aimed to identify cassava quality attributes preferred by users along the food chain, in order to provide breeders with criteria for prioritisation. Survey and consumer‐testing studies were conducted within Apac and Luwero districts in Uganda. Additionally, sensory evaluation by trained panellists was conducted to determine descriptors for assessing quality of boiled roots. Results revealed softness of boiled roots and in‐ground storability as key attributes influencing varietal preference besides high yield, non‐bitter roots, disease resistance, early maturity and drought resistance. For some attributes like in‐ground storability, preference differed significantly between locations and showed differentiation by gender. Local varieties were found to be superior in quality attributes. From sensory evaluation, twenty‐one descriptors associated with appearance, texture, taste and aroma of boiled roots were determined. Findings from this study are vital for breeders to adopt gender‐responsive approaches in order to develop varieties that meet the needs and preferences of end users.

## Graphical abstract

This study reveals that softness of boiled cassava roots and in‐ground storage‐ability as key attributes influencing varietal preference in addition to high yield, non‐bitter roots, disease resistance, early maturity and drought resistance

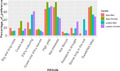

## Introduction

Cassava (*Manihot esculenta* Crantz) ranks as the sixth major crop after wheat, rice, maize, potato and barley and is an important source of food and income for over 800 million people globally (Lebot, 2009). The crop is grown throughout the tropics, with Africa alone accounting for more than half of total cassava produced worldwide (FAO, 2018). Additionally, it has been reported that cassava contains no gluten and that when its flour is mixed with egg white and extra‐virgin olive oil, it produces good bread quality comparable to the bread made from wheat, hence making gluten‐free cassava breads ideal for celiac patients (Pasqualone *et al*., 2010). The cassava crop is popular amongst smallholder farmers due to its inherent ability to give appreciable yields under sub‐optimal conditions and will survive in marginal conditions where other staple crops may fail completely (El‐Sharkawy, 1993; Ceballos *et al*., 2004; Kizito *et al*., 2007; FAO, 2013a). Cassava has flexible planting and harvesting times, and since it is clonally propagated by stem cuttings, there is no competition between use of the roots for propagation and for consumption (Nweke *et al*., 2002). Although all the parts of a cassava plant are used in one way or another, cassava is primarily grown for its enlarged starchy roots which can be utilised to make food, feed and industrial products (El‐Sharkawy, 2004; FAO, 2013a; Chiwona‐Karltun *et al*., 2015). The starchy roots provide consumers with a cheap source of calories (Nweke *et al*., 2002).

There are two broad categories in which cassava roots are consumed. Firstly, food products made from either freshly harvested roots, and secondly, flour is processed from roots and used to make different food products (Montagnac *et al*., 2009a; Bechoff *et al*., 2018). The form of consumption in part depends on typology and level of cyanogenic glucosides (FAO, 2013b), but also on local customs and preferences, that is even varieties with low cyanogens may be processed. For example, cassava to be consumed from freshly harvested roots, the varieties must have less than 50 mg kg^−1^ on fresh weight basis of the cyanogenic glucosides, while roots from varieties with high levels are processed prior to consumption in order to lower the level of glucosides (Cardoso *et al*., 2005; Montagnac *et al*., 2009b; FAO, 2013b).

It also suffices to note that cassava destined for human consumption must meet cooking and eating quality characteristics. However, eating quality is complex as it comprises of many different critical attributes, namely cooking time, texture, bitterness and flavour. Evidently, each of these attributes is almost a function of several sub‐components, which makes breeding for end user quality extremely challenging. Nonetheless, the importance of these attributes and their integration into breeding operations is getting prioritised.

Given the diverse forms in which cassava is consumed (Nweke *et al*., 2002), there is need to document the various quality attributes associated with each form. Once the attributes are identified, appropriate methodologies for measuring them can be developed and evaluated for breeding purposes. A study by Padonou *et al*. (2005) reported that results from sensory evaluation of boiled cassava for end user preferred attributes correlated well with instrumental characterisations. Cassava breeders have achieved genetic gains in improving cassava for most key agronomic attributes (Bechoff *et al*., 2018); however, there is limited documented information on the specific root quality attributes preferred by men and women who consume boiled roots, one of the major forms in which cassava is consumed. Consequently, some varieties that are developed, do not meet some of the key attributes preferred by the consumers, hence limiting adoption of the improved varieties which would potentially contribute to improvement of livelihoods and food security, particularly for poor resource households. Previously, studies have highlighted that cassava attributes preferred by users along the food chain influence the adoption of new varieties (Alene *et al*., 2013; Chiwona‐Karltun *et al*., 2015; Teeken *et al*., 2018). Thus, there is need to understand the cassava attributes preferred by end users to help cassava breeders determine the drivers of adoption and in turn be able to set breeding priorities that are more demand driven.

Accordingly, in this study, our objectives are as follows: (i) to determine the cassava quality attributes preferred by men and women end users; and (ii) to determine key descriptors that can be used in sensory evaluation of boiled cassava roots and then applied by breeders to improve consumer acceptability of new varieties.

## Materials and methods

This section is divided into two related objectives, namely (i) determination of qualities of cassava preferred by men and women based on survey and consumer‐testing and (ii) determination of sensory descriptors for sensory evaluation of boiled cassava, outlined hereafter.

### Survey and consumer‐testing

#### Research design and area

A descriptive survey employing mixed methods of data collection was conducted to understand the perceptions of men and women on food‐value attribute qualities of boiled cassava. Mixed methods of data collection were used to provide a more comprehensive approach to the research question (Almalki, 2016). Accordingly, data were collected from men and women who grow, process and/or consume boiled cassava. Luwero and Apac districts, respectively, located in central and northern Uganda, were selected owing to their high production (UBOS, 2010), diversity in processing and consumption of boiled cassava. These districts were selected following compilation of a state of knowledge on boiled cassava that involved undertaking literature reviews and conducting key informant interviews with critical stakeholders involved in cassava value‐chain.

#### Sampling and data collection

Our primary food product was boiled cassava. Accordingly, simple random sampling was employed to pre‐select sub‐counties and parishes using sampling frames provided by Luwero and Apac district agricultural officers. Subsequently, two sub‐counties were selected from each district and two parishes from each sub‐county. Participants in the study were purposively selected with an aim of enrolling approximately equal proportions of men and women of ages spanning from 18 to 70 years. The study questions were adapted or modified from a report by Forsythe *et al*. (2018) to design semi‐structured questionnaires and the guide for focus group discussions (FGDs).

Semi‐structured questionnaires were administered to twenty‐one men and nineteen women in Apac district, while in Luwero district, questionnaires were administered to seventeen men and twenty‐three women. All sampled respondents grow, process and/or consume boiled cassava. Furthermore, consumer‐testing was conducted in the study areas with focus on documenting culinary attributes of boiled cassava. The participants in consumer‐testing included fifty‐seven men and thirty‐seven women in Apac district, while in Luwero district, there were forty‐nine men and thirty‐eight women who evaluated boiled cassava from four to five varieties, and with descriptors generated from the data collected from the surveys. Luwero and Apac have divergent methods of processing boiled cassava. In Luwero, roots were peeled, cleaned in water, sliced into longitudinal sections and wrapped in banana leaves. The wrapped sections were placed in a saucepan containing a banana sheath and water (the banana sheath helps to stop the wrapped cassava sections from getting in contact with water) and then covered with two to three pairs of banana leaves. Similar to what people typically do, another saucepan was placed on top as a cover and then roots are cooked on a firewood stove for 1 h. In Apac, the sectioned cassava roots were placed in a saucepan prepared with water at about a quarter volume of the saucepan. Another saucepan was used as a cover and the roots were cooked for 45 min on a firewood stove. Adopting local cassava preparation and cooking methods, that is, steaming and boiling were considered to enable informed assessment of preferred quality attributes.

In preparation for consumer‐testing, caution was taken to select appropriate places which would be prepared with chairs and tables for comfort during testing and which allowed easy recruitment of men, women and youth. Upon seeking consent to participate, samples from all the four to five varieties were presented at ago since we were interested in the consumer's comment on the appearance and texture before tasting and evaluation. The enumerator administered the questionnaire to the participant as they tasted and evaluated the samples. To ensure content validity, the tools were reviewed by social science research professionals from National Crops Resources Research Institute (NaCRRI), the University of Greenwich, and the French Agricultural Research Centre for International Development to verify the representativeness and suitability of the questions. Ethical considerations were met by seeking the participants' consent to participate before commencing with the interview. Participants signed a consent form upon understanding the objective of the research and allowing to participate. In addition, the respondents were assured before the interviews began that any information linking results to individuals would be kept confidential.

### Determining key sensory descriptors for boiled cassava

Having learnt from consumer‐testing and surveys, there was need to establish standard descriptors that can be routinely used by sensory panellists to assess quality attributes during varietal development process by breeders. To this end, seven randomly selected plants per plot of a given variety were harvested and then randomly selected ten marketable roots taking care of root representation of the whole sample set. Soil and debri were removed from the roots before packing them in labelled bags prior to being transported to the laboratory for processing.

In the laboratory, the roots were washed to remove dirt and/or adhering soil debri. The distal and proximal ends of the roots were cut‐off and discarded, and thereafter, the remaining central portions of the roots were peeled and cut into equal root sections each measuring 6 cm long. Thereafter, fourteen to fifteen root sections were randomly sampled per clone, washed in clean tap water and wrapped in a banana leaf which was later tied with a banana fibre and a coloured string for ease of identification at serving point. The wrapped root sections were then cooked in a saucepan measuring at least 57.5 cm in diameter and height of 21.0 cm whose base had been laid with banana sheath and about 5000 mL of water added to it. The banana sheath is to stop water from getting in contact with the wrapped root sections to ensure cooking is only by steam.

Cooking was done on a gas cooker with four burners all set at maximum for 55 min. It was important to maintain the same water temperature and cooking time across all test samples. At the end of cooking, we turned off the gas cooker and allowed for 10 min of cooling at room temperature (about 23 °C) before we started serving the cooked samples to trained panellists who included seven males and six females for sensory evaluation. The panellists were purposively selected because they had been previously trained on how to undertake sensory evaluation of various food products from root, tuber and banana crops. Each panellist was provided a cup of drinking water (Rwenzori brand). At the time of serving boiled cassava to panellists, the plastic plates onto which the boiled cassava was served were labelled with unique labels corresponding to the test cassava clones.

Principally, one clone was served to the panellist at a time and requested to evaluate the sample based on their prior knowledge regarding appearance and texture both in mouth and touch; taste and aroma were also assessed. The panellists were given plain paper to write descriptors, after which a discussion was held with all panellists and the team facilitating the activity. The panellists were requested to highlight the key terminologies they had used to evaluate the attributes of cooked cassava. The highlighted descriptors were written on flip charts that were pinned on the wall of the training room so that everybody could easily follow the discussion. The highlighted descriptors were carefully grouped into categories such as texture by hand, aroma, appearance, taste and texture by mouth. In the end, after a series of discussions and evaluating a number of cassava clones, a catalogue of descriptors for boiled cassava was developed Table 1.

**Table 1:** Descriptors for sensory evaluation of boiled cassava roots with their respective definitions and approaches on how they can be measured

Category	Descriptor	Definition	How to measure	Scale
Appearance	Yellow	Colour of the surface of the sample from light yellow to bright yellow	Observe the surface of the sample, and evaluate the intensity of the colour, homogeneity, translucency, and surface smoothness	0: Non‐yellow 10: Bright yellow
	White	Colour of the surface of the sample from cream to bright white		0: Cream 10: Bright white
	Homogeneity of colour	Uniformity of colour of the surface of the sample		0: Heterogeneous 10: Homogeneous
	Surface Smoothness	Absence of rough parts, lumps or holes, fibre lines, and ridges especially in a way that is pleasant and attractive to touch		0: Very rough 10: Very smooth
Texture in mouth	Softness	Mechanical textural attribute relating to the force required to achieve a given deformation, penetration or breakage of a product	Put a part of the sample in the mouth, evaluate during the first bite (between molars) how hard the sample is	0: Soft 5: Firm 10: Hard
	Moisture	Perception of moisture content of a food by the tactile receptors in the mouth and also in relation to the lubricating properties of the product	Put a part of the sample in the mouth, chew and evaluate the quantity of water within the sample	0: Dry 10: Moist
	Smoothness	Geometrical textural attribute relating to lack of presence of particles in a product	Put a part of the sample in the mouth, chew it and after ten chews, evaluate between tongue and palate the number and the size of the particles	0: Lumpy 5: Grainy 10: Smooth
	Fibrousness	Presence of threads/fibres as you chew	Put a part of the sample in the mouth, chew it and after ten chews, evaluate between tongue and palate the presence and amount of fibre you feel	0: Non fibrous 5: Medium intensity 10: Very fibrous
	Mealiness	Characterised by being dry and crumbly like boiled egg yolk	Put a part of the sample in the mouth, chew it and after five chews, evaluate between tongue and palate the extent of dry and crumbly	0: Not mealy 5: Medium mealiness 10: Very mealy
	‘*Kiwuta*’ (Physiological state of starch immobilisation before harvest)	Characterised by any of the following: oozes upon squeezing, crunchy like raw carrot, remains raw after cooking, glassy appearance, tasteless, sweeter than original, usually have off‐flavour	Put a part of the sample in the mouth, chew it and after five chews, evaluate between tongue and palate the presence of the given characteristics	YES/NO
	Stickiness	Mechanical textural attribute relating to the force required to remove material that sticks to the mouth	Put a part of the sample in the mouth, chew it and evaluate: amount of product adhering on/in the teeth after product mastication and the force required to remove product completely from the palate, using the tongue, after complete compression of the sample between tongue and palate	0: Non sticky 10: Sticky
Texture by touch	Mealiness	Characterised by being dry and crumbly like boiled egg	Put a part of the sample between thumb and index fingers and try to press it, evaluate the dryness and ability of product forming into powder	0: Not mealy 5: Medium mealiness 10: Very mealy
	Moldability	Mechanical textural attribute relating to the degree to which a substance can be deformed before it breaks	Try to make a ball (agglomerate) of the sample and evaluate how easy it is to deform or break the sample	0: Crumbly 10: Moldable
	Stickiness	Mechanical textural attribute relating to the force required to remove material that sticks to the mouth	Put a part of the sample between the thumb and index fingers and using tapping motions, evaluate the amount of product adhering on them	0: Non sticky 10: Sticky
Taste	Sweetness	Basic taste produced by dilute aqueous solutions of natural or artificial substances such as sucrose	Put a part of the sample in the mouth and evaluate the intensity of taste of sugar	0: No sweetness 3: Low intensity 6: Medium intensity 10: High intensity
	Bitterness	Basic taste produced by dilute aqueous solutions of natural or artificial substances such as quinine	Put a part of the sample in the mouth and evaluate the intensity of bitterness	0: No bitterness 3: Low intensity 6: Medium intensity 10: High intensity
	Bitter after taste	Feeling of slight bitterness after swallow	Put a part of the sample in the mouth and evaluate whether bitter after swallowing	YES/NO
Aroma	Cassava	Aroma of the steamed cassava	Put a part of the product and by retro‐olfaction evaluate the presence and the intensity of this specific aromas	0: Low intensity 5: Medium intensity 10: High intensity
	Roasted Cassava	Aroma of roasted cassava		YES/NO
	Yam	Aroma of yam		YES/NO
	Sweet Potato	Aroma of sweet potato		YES/NO

### Statistical analyses

Content analysis was employed to analyse qualitative data collected from the surveys and consumer‐testing (Elo & Kyngäs, 2008). The generated responses were carefully read to develop appropriate codes representing similar themes of data. The themes generated were disaggregated by sex and location to understand the gender‐related perceptions of boiled cassava. Consequently, the generated themes, along with the quantitative data for the other variables that were prior coded were analysed using descriptive statistics using the statistical package for social scientists software (SPSS, 2012) to obtain the percentages of the responses to each variable. A chi‐square test of independence was used to determine whether the relationships between the given variables are significant.

## Results and discussion

### Cassava attributes preferred by men and women within Apac and Luwero districts

During the survey, men and women cassava end users were requested to mention the general attributes they preferred about cassava. Results (Table 2) indicated softness of boiled roots and in‐ground storability in addition to high fresh yield, non‐bitter roots, disease resistance, early maturity and drought resistance are the predominant attributes preferred by end users. This information provides new insights to breeders involved in developing new cassava varieties that meet the needs of all end users, including consumers (Acheampong *et al*., 2018). Multiple preferences necessitate: (i) development of robust trait assessment methods and (ii) use of appropriate selection indices that take into consideration trait importance and heritability (Acquaah, 2012). The preference for some of the attributes such as high fresh root yield and softness of boiled roots were not significantly different (*P*‐value > 0.05) between men and women, and across different locations (Table 2). Within a given district, the percentage of men who prefer certain attributes is not significantly higher (*P*‐value > 0.05) than that of women except for in‐ground storage ability attribute for which men had a significantly higher (*P*‐value < 0.05) percentage than women (Table 2). In contrast, the percentage of end users who preferred sweet/non‐bitter roots, non‐diseased roots, early maturity and in‐ground storage ability was significantly different (*P*‐value < 0.05) between Apac and Luwero, there were no significant differences (*P*‐value > 0.05) between the two districts for the other attributes (Table 2). The proportion of women who prefer non‐diseased cassava roots is significantly higher (*P*‐value < 0.05) in Apac than in Luwero, while the percentage of women who prefer early maturing and drought‐resistant varieties is significantly higher (*P*‐value < 0.05) in Luwero than Apac (Table 2). Additionally, results indicate that more men in Luwero prefer in‐ground storability of roots than men in Apac. Gender differentiated trait preferences have also been reported in previous studies (Teeken *et al*., 2018). The implication of this finding is that breeders may need to adopt gender‐responsive breeding approach such as breeding for the attributes preferred by men and women end users across varying locations. Men and women in different locations may have varied gender‐roles, and these may influence their needs and preferences in a particular crop (Bezner Kerr, 2017).

**Table 2:** Agronomic and quality attributes of cassava along the food chain preferred by men and women who participated in the survey in Apac and Luwero

Attribute^a^	Apac district			Luwero district			District			Women			Men		
	Men	Women	*P*‐value	Men	Women	*P*‐value	Apac	Luwero	*P*‐value	Apac	Luwero	*P*‐value	Apac	Luwero	*P*‐value
High yield	66.7	84.2	ns	70.6	73.9	ns	75.0	72.5	ns	84.2	73.9	ns	66.7	70.6	0.796
Not bitter	71.4	73.7	ns	52.9	47.8	ns	72.5	50.0	0.039*	73.7	47.8	ns	71.1	52.9	0.240
Not diseased	71.4	84.2	ns	47.1	43.5	ns	77.5	45.0	0.010*	84.2	43.5	0.007**	71.4	47.1	0.126
Early maturing	33.3	15.8	ns	52.9	60.9	ns	25.0	57.5	0.003**	15.8	60.9	0.003**	33.3	52.9	0.224
Cooks soft	19.0	36.8	ns	11.8	17.4	ns	27.5	15.0	ns	36.8	17.4	ns	19.0	11.8	0.540
Drought resistant	19.0	5.3	ns	17.6	34.8	ns	12.5	27.5	ns	5.3	34.8	0.020*	19.0	17.6	0.912
In‐ground storability	4.8	0.0	ns	41.2	13.0	0.042*	2.5	25.0	0.003*	0.0	13.0	ns	4.8	41.2	0.006**
Green and shiny leaves	14.3	15.8	ns	11.8	8.7	ns	15.0	10.0	ns	15.8	8.7	ns	14.3	11.8	0.819
Big and long roots	19.0	10.5	ns	11.8	0.0	ns	15.0	5.0	ns	10.5	0.0	ns	19.0	11.8	0.540
Not fibrous	9.5	5.3	ns	5.9	4.3	ns	7.5	5.0	ns	5.3	4.3	ns	9.5	5.9	0.679

ns, not significant.

^a^ The preferences of men and women are expressed as percentages

* *P*‐value < 0.05.

** *P*‐value < 0.01.

End users in Apac prefer Bao, NAROCASS 1 and NASE 14, varieties for boiled form of consumption, whereas in Luwero the most preferred varieties are NASE 14 and TME 14 (Table 3). Information gathered from the FGDs indicated that farmers had multiple reasons for preferring a given variety: *‘Bao is resistant to disease, cooks soft food, not bitter, has nice aroma, it is high yielding and easy to peel’ men FGD in Apac*. In addition to NASE 14 and NAROCASS 1 varieties being consumed in form of boiled cassava roots by end users in Apac, the varieties are also predominantly used for brewing *Mogamoga* (local brew), an activity mainly done by women who claim that NAROCASS 1 produce tasty and much brew compared to other varieties. Despite Bao being the most preferred variety in Apac, it was not mentioned as one of the preferred varieties in Luwero. Previously, a study by Iragaba *et al*. (2020) also found that smallholder farmers in different districts grow different varieties, a phenomenon that could tag varietal preferences to specific communities owing to relative ease of access of planting materials within a community as compared to ease of access between communities. With continued engagement of end users in the breeding process and increasing access to improved seed, such disparities could be reduced. Differential preferences for varieties may call for targeted breeding to respond to the needs of men and women in a particular locality. It was observed that people have preference for some varieties because they yield multiple products e.g. in one of the FGDs NASE 14 was preferred: ‘*We use it prepare boiled cassava, mashed cassava (mogo myeno), cassava flour, kalo (cassava thick paste), and produces a lot of local brew’ women FGD in Apac*. Information collected from a women FGD in Apac district indicated that NAROCASS 1 was preferred: ‘*It is high yielding, the stems have ready market and makes very strong local brew which has a nice aroma’*.

**Table 3:** Percentages of men and women who preferred certain varieties for boiled cassava did not significantly differ in Apac and Luwero districts

Variety^a^	Apac			Luwero		
	Men (%)	Women (%)	*P*‐value	Men (%)	Women (%)	*P*‐value
NASE 14	33.3	36.8	ns	70.6	52.2	ns
Bao	76.2	73.7	ns	0.0	0.0	
NAROCASS 1	38.1	36.8	ns	5.9	8.7	ns
TME 14	0.0	0.0		17.6	34.8	ns
Bukalasa	0.0	0.0		5.9	8.7	ns
Kakuta kamyufu	0.0	0.0		11.8	4.3	ns
NASE 19	4.8	5.3	ns	0.0	0.0	
Katebe	0.0	0.0		0.0	4.3	
Kayumba	0.0	0.0		0.0	4.3	
Odyek Leo	4.8	0.0		0.0	0.0	
Tim Tim	4.8	0.0		0.0	0.0	
Kawuki	0.0	0.0		0.0	4.3	

^a^ Varieties with zero per cent preference means that men or women in a given district did not mention the given variety as one of the varieties they preferred for boiled cassava. ns means there were no significant differences at *P*‐value < 0.05. Chi‐square tests were not computed for varieties that had low proportion of preference.

**Figure 1:**
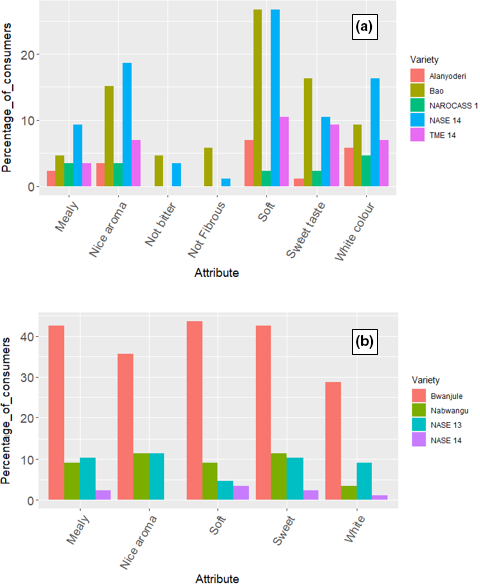
Most preferred varieties and their attributes during consumer‐testing in Apac (a) Luwero (b).

Consumer‐testing results indicate that most of the attributes preferred by users are the same as those we identified during the survey (Fig. 1). The fact that even with a different group of participants, similar attributes were raised, points out the weight end users attach to the mentioned attributes within the respective localities. We also observed local varieties such as Bao and Bwanjule were the most preferred varieties meeting most preferences of users (Fig. 1). Information gathered from FGDs indicated that these local varieties are most preferred because they produce white, soft, sweet and aromatic boiled cassava. Similar to our finding, previous studies also reported that local varieties were more preferred (Alene *et al*., 2013; Nakabonge *et al*., 2018). These findings justify the need to use selected local varieties as progenitors in our efforts of implementing demand‐led breeding in an effort to breed varieties that meet user preferences along the food chain. This is particularly important intervention that can contribute to increase acceptability of new improved varieties.

Participants in the consumer‐testing exercise were also asked how often they consumed boiled cassava. Results indicated that most consumed cassava on a daily basis or several times a week (Table 4). This result is concordant to earlier studies which documented cassava as a key staple crop in both urban and rural communities for majority of people in Uganda (Nweke *et al*., 2002; Otim‐Nape *et al*., 2005). This finding confirms the major role cassava plays towards sustaining livelihoods of men and women in study areas. It is such impacts that breeding programmes should desire to attain and upscale.

**Table 4:** Frequency of how often men and women consume boiled cassava in Apac and Luwero districts

How often consumers eat boiled cassava	Apac district		Luwero district	
	Men (%)	Women (%)	Men (%)	Women (%)
Every day	63.2	67.6	22.4	23.7
Several times a week^a^	31.6	18.9	49.0	39.5
Once a week	1.8	5.4	12.2	15.8
Once a month	1.8	0.0	0.0	0.0
Others	1.8	8.1	16.3	21.1

^a^ Several times a week refers to scenarios where men and women responded they consumed boiled cassava more than once a week

### Sensory descriptors for boiled cassava

In addition to the survey and consumer‐testing, sensory evaluation was conducted to develop descriptors which could be used in routine assessment of quality boiled cassava of new cassava clones developed by the breeding programmes. For each of the major preferred attributes of boiled cassava identified by end users, we developed descriptors and a standardised methodology by which they can be assessed by panellists (Table 1). Accordingly, twenty‐one descriptors associated with appearance, texture, taste and aroma were developed (Table 1). Subsequently, the developed descriptors could be used in developing more robust and high‐throughput phenotyping technologies such as use of near infra‐red spectroscopy (NIRS). Previous studies have indicated that consumer‐testing and sensory evaluation results can have high correlations with instrument characterisation (Padonou *et al*., 2005; Iragaba *et al*., 2019). Additionally, sensory descriptors developed in this study could be used by breeders in assessing the quality of the cassava breeding lines before they are released to the end users. Currently, efforts are devoted towards development of reproducible methods for measuring attributes with appreciable genetic variation in our cassava breeding populations. The first priority is softness of boiled roots that we propose to measure using near infra‐red spectroscopy (NIRS), all leveraging on previous work (Iragaba *et al*., 2019).

## Conclusions

Data sets presented in this paper have two major findings. Firstly, that men and women ranked softness of boiled roots and in‐ground storability in addition to high fresh yield, non‐bitter roots, disease resistance, early maturity and drought resistance as the most preferred quality attributes about cassava. Exceptionally, the preference for soft boiled cassava roots was not significantly different (*P*‐value > 0.05) between men and women both within a given district and across different districts, whereas in‐ground storability of cassava roots was more preferred by men in Luwero district. In designing breeding programmes, there is a need to consider the differentiated preferences in order to develop varieties that meet the needs, aspirations and preferences of both men and women. Most consumers indicated that they eat cassava on a daily basis. This points out the importance of the crop in the livelihoods of men and women in study areas. End users in the food chain for boiled cassava indicated an array of preferred attributes. This information is critical for breeders in developing new varieties that meet the needs and preferences of users along the food chain. Breeding for multiple attributes calls for systematic attribute prioritisation during breeding work. Use of selection indices can optimise the weight given to each of the attributes and increase the likelihood of adoption of new varieties. The varieties found to have desirable attributes preferred by end users could be used to enrich breeders' germplasm. Secondly, in this paper, the twenty‐one sensory descriptors determined could be used by breeders in assessing the quality of the cassava breeding lines before they are released to the end users.

## Conflict of interest

The authors declare no conflict of interest in this work.

## Author contribution

**Paula Iragaba:** Conceptualization (equal); Data curation (equal); Formal analysis (lead); Methodology (equal); Writing‐original draft (lead); Writing‐review & editing (lead). **Sophia Hamba:** Conceptualization (equal); Data curation (equal); Formal analysis (equal); Methodology (equal); Writing‐original draft (equal); Writing‐review & editing (equal). **Ritah Ann Nanyonjo:** Conceptualization (equal); Data curation (equal); Formal analysis (equal); Methodology (equal); Writing‐original draft (equal); Writing‐review & editing (equal). **Michael Kanaabi:** Conceptualization (equal); Data curation (equal); Formal analysis (equal); Methodology (equal); Writing‐original draft (equal); Writing‐review & editing (equal). **Ephraim Nuwamanya:** Conceptualization (equal); Data curation (equal); Formal analysis (equal); Methodology (equal); Writing‐original draft (equal); Writing‐review & editing (equal). **Doreen Mpamire:** Data curation (equal); Formal analysis (equal); Methodology (equal); Writing‐original draft (equal); Writing‐review & editing (equal). **Nicholas Muhumuza:** Data curation (equal); Formal analysis (equal); Methodology (equal); Writing‐original draft (equal); Writing‐review & editing (equal). **Elizabeth Khakasa:** Conceptualization (supporting); Data curation (equal); Formal analysis (equal); Methodology (equal); Writing‐original draft (equal); Writing‐review & editing (equal). **Hale Ann Tufan:** Conceptualization (equal); Funding acquisition (equal); Investigation (equal); Methodology (equal); Supervision (equal); Writing‐review & editing (equal). **Robert Sezi Kawuki:** Conceptualization (equal); Data curation (equal); Formal analysis (supporting); Funding acquisition (equal); Investigation (equal); Methodology (equal); Project administration (lead); Resources (equal); Supervision (lead); Validation (equal); Visualization (equal); Writing‐original draft (supporting); Writing‐review & editing (supporting).

## Ethical guidelines

This study was approved by the National Research Ethics Committee. Research teams obtained ethical approval prior to the fieldwork. Participants were informed about the study objectives, they could stop the interview at any point. Consent from sensory panellists and from consumers participating in this study was obtained and the research respected the rules of voluntary participation and anonymity. Food samples were prepared according to good hygiene and manufacturing practices.

## Data Availability

The data that support the findings of this study are available on request from the corresponding author. The data are not publicly available due to privacy or ethical restrictions.
